# Supplementary material for: Comparative multiomics analysis of cell physiological state after culture in a basket bioreactor
Source: Sci Rep. 2022 Nov 23;12:20161. doi: 10.1038/s41598-022-24687-4 (PMC9686226; doi:10.1038/s41598-022-24687-4)
Supplement: Supplementary file 1 — Supplementary Information 1. [file 41598_2022_24687_MOESM1_ESM.zip › raw data/Proteomic raw data/1.data_control/bar.pdf]

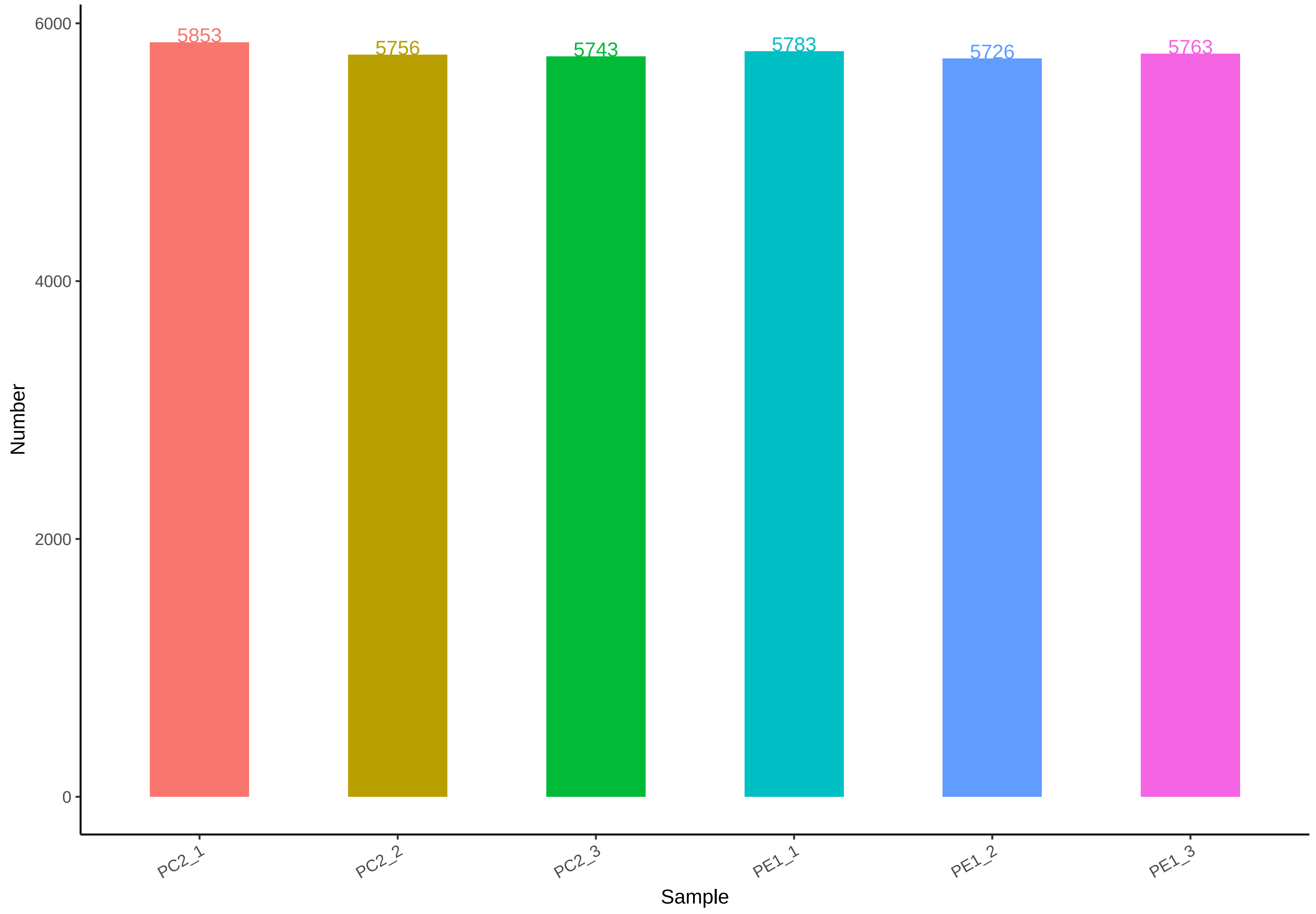

5853
5756
5743
5783
5726
5763
6000
4000
2000
0
Number
PC2_1
PC2_2
PC2_3
PE1_1
PE1_2
PE1_3
Sample
